# Automated Segmentation of Augmented Bone After Transalveolar Sinus Floor Elevation Using Deep Learning

**DOI:** 10.1016/j.identj.2026.109468

**Published:** 2026-03-06

**Authors:** Kexin Yang, Wenjun Duan, Wangtao Lu, Zheyuan Sun, Rongan Li, Jiakang Yang, Baixiang Wang

**Affiliations:** a Stomatology Hospital, School of Stomatology, Zhejiang Provincial Clinical Research Center for Oral Diseases, Key Laboratory of Oral Biomedical Research of Zhejiang Province, Engineering Research Center of Oral Biomaterials and Devices of Zhejiang Province, Zhejiang University School of Medicine, Cancer Center of Zhejiang University, Hangzhou, China; b State Key Laboratory of Industrial Control Technology and Institute of Cyber-Systems and Control, Zhejiang University, Hangzhou, China

**Keywords:** TSFE, Bone augmentation, Automatic segmentation, Deep learning, Artificial intelligence, UNETR++

## Abstract

This study aimed to evaluate the performance of deep learning models for segmenting the augmented bone following transalveolar sinus floor elevation (TSFE). Cone-beam computed tomography (CBCT) data from 103 patients undergoing TSFE, acquired at preoperative (T0) and immediate postoperative (T1) were retrospectively analysed. Four deep learning models (UNETR++, Swin Transformer, U-Net, 3D-VNet) were trained and validated for segmenting the augmented bone. Performance was assessed using the Dice similarity coefficient (DSC), intersection over union (IoU), sensitivity, precision, 95% Hausdorff Distance (HD95), and accuracy. UNETR++ demonstrated the best performance, with an average DSC of 0.8477, IoU of 0.7356, sensitivity of 0.8337, precision of 0.8622, HD95 of 0.9234 mm, and accuracy of 0.8730. UNETR++ segmentations exhibited excellent reproducibility compared with manual segmentation. The automated segmentation process significantly reduced measurement time to 14.96 ± 2.57 seconds. Deep learning models, particularly UNETR++, provide an accurate and efficient method for segmenting augmented bone after TSFE.

## Introduction

Transalveolar sinus floor elevation (TSFE) is a commonly used surgical strategy for implant restoration in cases of insufficient bone volume in the posterior maxilla.^1^ TSFE involves elevating the maxillary sinus membrane at the implant site and grafting bone material, without requiring flap reflection or bone window creation.^2^ Consequently, TSFE results in less surgical trauma and a milder postoperative response.^3^ However, the long-term effects of this approach still require further evaluation. Bone resorption is a common issue during the postoperative period and healing phase, which can affect implant stability and even lead to implant failure.^4^ This issue is particularly prominent for TSFE. As the surgery is typically performed blindly, it is difficult for clinicians to precisely control the amount of bone graft intraoperatively or to directly observe the immediate bone augmentation effect post-surgery.^5^ This initial uncertainty makes it extremely difficult to effectively monitor the subsequent dynamic process of bone resorption. Therefore, precise and objective quantification of the augmented bone volume and its dynamic changes postoperatively is not merely for academic evaluation. More importantly, it provides a critical basis for clinical decisions, such as predicting the long-term stability of the implant, determining the optimal timing for prosthetic loading, and identifying high-risk cases of potential failure.^6^ This precision is essential for improving surgical success rates and ensuring long-term patient outcomes.

Currently, bone volume is primarily measured using two-dimensional (2D) methods, which only assess height on panoramic radiographs or CT cross-sections, providing solely planar information. Compared to traditional methods, three-dimensional (3D) measurement techniques are time-consuming and complex.^7^ Moreover, the lack of standardized measurement criteria among different clinicians poses a significant challenge for accurate assessment. A stable method for augmented bone volume measurement would also help standardize clinical research. To achieve this, accurate augmented bone segmentation is a prerequisite. Current manual and semi-automated segmentation methods are labour-intensive, heavily reliant on operator experience, and susceptible to errors.^8^^,^^9^ These bottlenecks not only hinder the precise evaluation of postoperative outcomes for TSFE but also limit the standardization of related clinical research.

In the field of dentistry, deep learning techniques have been widely applied across various areas, including clinical diagnosis and decision, preoperative planning, postoperative analysis, and prognostic prediction.^10^, ^11^, ^12^ For instance, deep learning has demonstrated high precision in image recognition and diagnosis, showcasing its accuracy and efficiency in the segmentation of maxillofacial anatomical structures.^13^^,^^14^ Previous studies have conducted segmentation analyses of augmented bone following lateral sinus floor elevation (LSFE), but research specifically evaluating TSFE remains limited.^15^ In such cases, volume measurements may be subject to significant deviations, potentially affecting the segmentation performance of deep learning models. Therefore, it is necessary to further assess the effectiveness of deep learning models in segmenting augmented bone following TSFE.

Specifically, this study focuses on comparing the performance of various deep learning architectures, including transformer-based models (UNETR++ and Swin Transformer) and convolutional neural networks (CNN)-based models (U-Net and 3D V-Net). Among the numerous segmentation methods, U-Net, inspired by fully convolutional networks (FCNs) and encoder-decoder architectures, has emerged as a cornerstone model in biomedical image segmentation, widely applicable across various imaging modalities.^16^ To address the limitation of 2D U-Nets in capturing Z-axis (depth) context for slice-processed 3D medical images, 3D V-Net was developed. This utilize 3D convolutions for faster and more precise 3D segmentation.^17^ More recently, Transformer models, originating from natural language processing (NLP) and leveraging self-attention for robust global context modelling, have shown remarkable success in computer vision, particularly in medical image segmentation tasks when incorporating mechanisms like spatial attention.^18^ However, conventional transformer-based methods often overlook crucial channel-wise feature dependencies and can be computationally expensive, especially when processing 3D medical images.^19^ To address these limitations, UNETR++ introduces the novel efficient paired attention (EPA) module.^20^ This module effectively captures both spatial and channel-wise information by jointly learning these representations through a unified attention mechanism. By projecting the key (K) and value (V) matrices of the self-attention operation into a lower-dimensional space, EPA reduces computational complexity while enhancing the representation capability for both spatial and channel-wise features.

This study aims to explore the potential of using deep learning techniques on postoperative CBCT scans for the automated segmentation of augmented bone following TSFE. This will involve training and validating deep learning models to accurately segment augmented bone, thereby improving the efficiency and consistency of volumetric analysis. This research is expected to provide clinicians with an efficient and reliable tool for quantitative analysis, overcoming the limitations of traditional measurement methods. The goal is to advance the postoperative evaluation of TSFE toward greater precision and standardization, ultimately improving the long-term success rate of implant restorations.

## Method

This study was designed as a retrospective analysis and is reported in accordance with the STARD guidelines. Informed consent was waived. The imaging records of patients who underwent maxillary sinus floor elevation via the transalveolar approach at the Zhejiang University School of Medicine, Affiliated Stomatology Hospital, from January 2017 to December 2024, were reviewed. The study adhered to the ethical principles outlined in the Declaration of Helsinki for clinical research involving human subjects and was approved by the Ethics Committee (Approval No. 2024-03-222).

### Data

This retrospective study involved 103 patients who underwent TSFE with simultaneous implant placement. All patients had a preoperative (T0) and postoperative(T1) CBCT scan. The study cohort comprised patients aged 20 to 79 years, with a gender distribution of 59 males (57.3%) versus 44 females (42.7%). The cohort was randomly partitioned at the patient level into a training set (82 patients) and a test set (21 patients) (Table 1). All corresponding postoperative CBCT scans for the patients within these sets were registered to enable accurate segmentation and volumetric comparison. CBCT scans were obtained using the NewTom 3G CBCT (QR) system, which has a field of view (FOV) of 15 × 12 cm and an exposure setting of 110 kV.

**Table 1:** Description of CBCT dataset.

		Training Set	Test Set
Patients number		82	21
Female		46	13
Male		36	8
Age (years)		49.0 ± 15.75 (range: 20-79)	46.57 ± 13.15 (range: 26-71)
Image dimensions (voxel)	x	484.53 ± 51.69	462.92 ± 54.64
	y	478.38 ± 55.68	452.46 ± 60.30
	z	389.51 ± 59.354	353.38 ± 62.86
Voxel size (mm)		0.25 (18 cases)	0.25 (5 cases)
		0.29 (14 cases)	0.29 (5 cases)
		0.30 (46 cases)	0.3 (9 cases)
		0.35 (4 cases)	0.35 (2 cases)

Inclusion criteria for the study required that the CBCT scans had a sufficiently wide FOV to encompass the entire augmented bone area. Only scans free from motion artifacts and from patients without acute maxillary sinusitis or sinus cysts were included. Patients exhibiting significant image distortion or any of these conditions were excluded to maintain data quality and avoid confounding factors. All patients underwent TSFE with simultaneous implant placement, and the graft material used for sinus floor augmentation was Bio-Oss Collagen (Geistlich, Switzerland), a bovine-derived hydroxyapatite-based bone graft substitute commonly used in sinus lift procedures.^21^

### Data preprocessing

All annotations were performed using 3D Slicer (version 5.2.2) following a workflow comprising three stages.^22^ First, the initial approximation of the augmented bone boundary was delineated using the contour evolution tool. Subsequently, the region of interest was manually refined on each individual slice based on anatomical landmarks. Finally, the annotations were examined in the 3D view to confirm spatial continuity and accuracy. These ground truth annotations were created by two trained surgeons who were blinded to all clinical information. To ensure consistency, they first established a consensus annotation guideline by collaboratively resolving disagreements on an initial subset of 20 cases. For the remaining dataset, the surgeons divided the annotation task, and each surgeon’s work was subsequently cross-validated and finalized by the other. If a consensus could not be reached, a third expert was consulted for final arbitration.

To enable standardized extraction of the region of interest (ROI), postoperative CBCT scans were registered to their corresponding preoperative scans, which served as a fixed reference. Using the Elastix toolbox, the registration process involved a sequence of rigid, affine, and non-rigid B-spline transformations to establish a common anatomical coordinate system.^23^^,^^24^ Registration was performed between the original preoperative CBCT and postoperative CBCT scans to ensure spatial consistency. After registration, a ROI was extracted from the registered postoperative CBCT scan. This ROI was centred on the implant, encompassing the entire bone graft material and the surrounding bone. The extraction process was guided by the precise location and extent of the augmented bone, using it as a reference to define the boundaries of the ROI. This approach ensured that the ROI captured all relevant information related to graft integration and surrounding bone remodelling. To standardize the data, the ROI was cropped into a fixed 96 × 96 × 64 voxel size, centred around the implant. This size was chosen to ensure that it fully covers all the augmented bone and the implant in the dataset while excluding unnecessary areas.

Additionally, all images were normalized to a range of 0 to 255 to standardize intensity values across the dataset. This normalization helped ensure consistency in the data for further analysis and comparison, optimizing computational efficiency while preserving essential details for accurate evaluation of augmented bone.

For data augmentation, several strategies were applied, including rotation within a range of ±15° and flipping along the x, y and z axes. Additionally, random noise was introduced during the training process to enhance generalization capability.

### Deep learning

#### Model architecture

The input to the segmentation model consists of a standardized ROI extracted from each aligned postoperative CBCT scan. The output is the segmentation of augmented bone, which are meticulously annotated by clinical experts to serve as the ground truth for training and evaluation. Within this framework, the UNETR++ model was employed as the segmentation framework, with a transformer-based backbone. The network architecture is illustrated in Fig. 1a, Fig. 1b. The encoder consists of four stages, with the first stage including a patch embedding layer that divides the volumetric input into 3D patches. Each efficient parallel attention (EPA) block comprises two attention modules that encode spatial and channel-wise information using a shared keys-queries scheme, effectively learning rich spatial-channel feature representations. The encoder stages are connected to the decoder stages via skip connections to merge outputs at different resolutions. This design helps recover spatial information lost during down sampling operations, enabling more accurate predictions.

**Fig. 1a:**
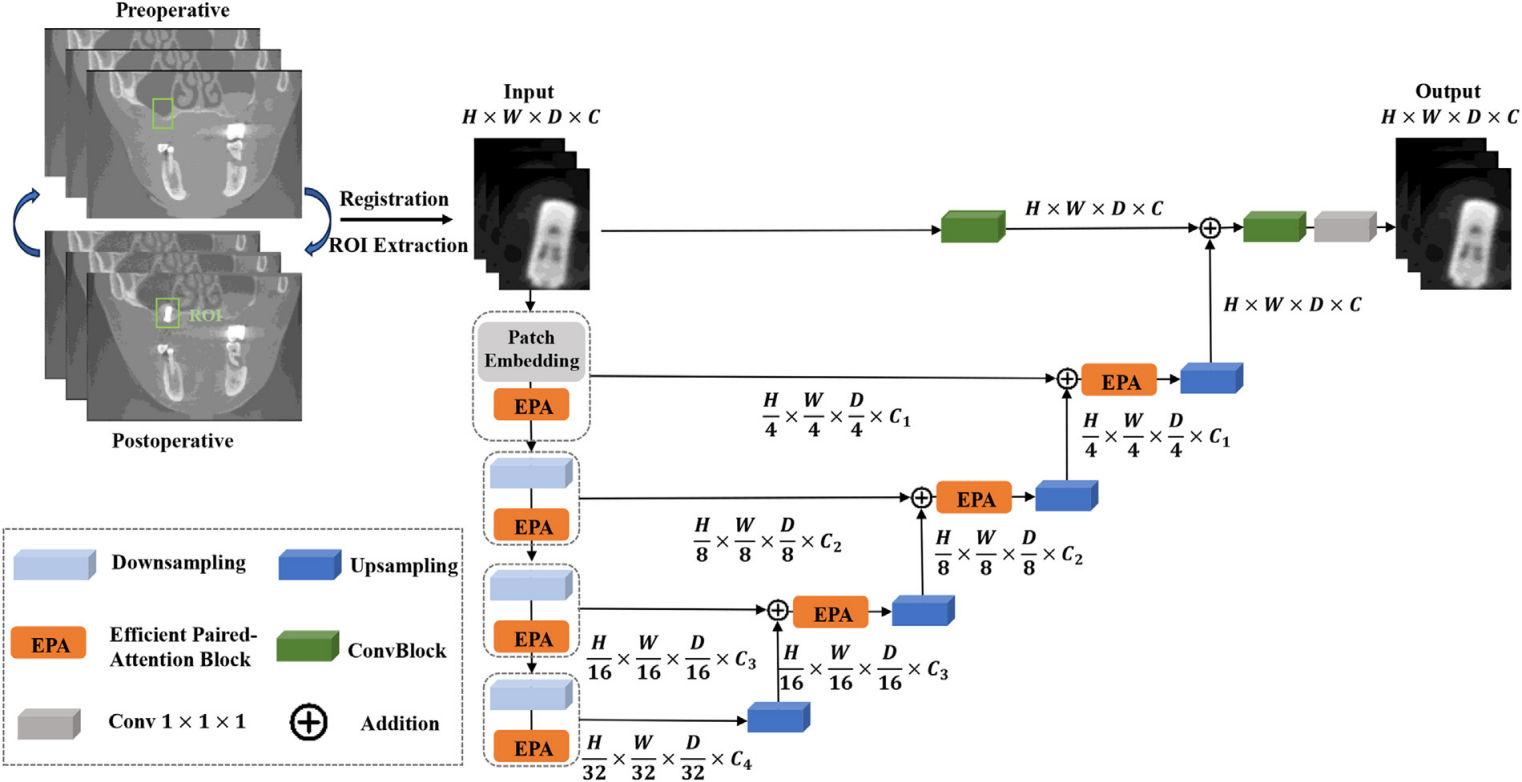
Architecture of the UNETR++ model.

**Fig. 1b:**
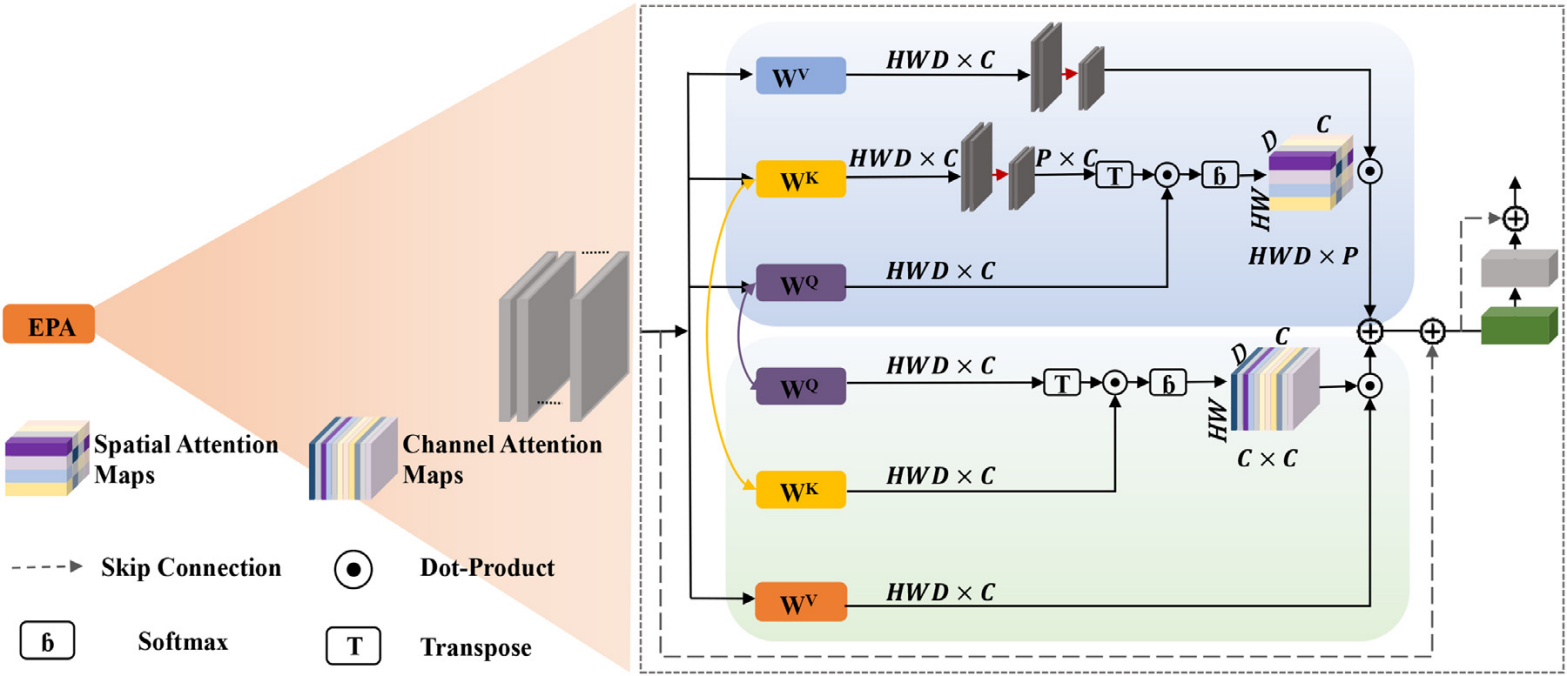
Architecture of the EPA module.

Similar to the encoder, the decoder also consists of four stages. Each decoder stage includes an up sampling layer that doubles the resolution of feature maps using transposed convolutions, followed by an EPA block (except for the final decoder stage). The number of channels is reduced by a factor of two between every two consecutive decoder stages. The output of the final decoder stage is fused with convolutional feature maps to recover spatial information and enhance feature representations. The resulting output is then passed through 3 × 3 × 3 and 1 × 1 × 1 convolutional blocks to generate the final voxel-wise mask prediction.

The EPA module performs efficient global attention, effectively capturing rich spatial-channel feature representations, as illustrated in Figure 1b. It consists of a spatial attention module and a channel attention module. The spatial attention module reduces the complexity of self-attention from quadratic to linear, while the channel attention module effectively learns interdependencies among channel feature maps. The EPA module leverages a shared keys-queries scheme between the two attention modules to facilitate information exchange, resulting in better and more efficient feature representations. This improvement is likely due to the shared keys and queries enabling the learning of complementary features, while distinct value layers are used for each attention module. For comparison, the architectures of Swin Transformer, U-Net, and 3D-VNet are presented in Figures S1-S3, respectively.

The workflow begins with the registration of preoperative and postoperative CBCT scans. Key components of the architecture include down sampling and up sampling paths, efficient paired-attention blocks, convolutional blocks (convblock), 1 × 1 × 1 convolutional layers (conv1 × 1 × 1), and addition operations. These elements work together to enhance feature extraction and segmentation accuracy.

It effectively captures rich spatial-channel feature representations through spatial attention maps and channel attention maps. The module also incorporates skip connections, dot-product operations, softmax functions, and transpose operations to refine the attention mechanism and improve the model’s ability to focus on relevant features.

#### Training details

The Adam optimizer was employed with an initial learning rate of 0.0001, which decayed at a rate of 3e-5. Training was extended to 1000 epochs to guarantee full convergence across all architectures. The final evaluation was conducted on the independent test set using the stabilized model weights to accurately reflect generalization performance. All experiments were conducted on an NVIDIA RTX A6000 GPU with 50 GB of memory. The implementation was based on PyTorch (version 1.11.0+cu113) with Python 3.8 and CUDA 11.3.

#### Loss function

The loss function integrates the advantages of the soft dice loss and cross-entropy loss, where$\;Y_{v\;}$and $P_{v}$ represent the ground truth and predicted probability at voxel v, respectively, and V denotes the total number of voxels.

$$L\left(Y,P\right)=1-\left(\frac{2*\sum_{v=1}^{V}Y_{v}\cdot P_{v}}{\sum_{v=1}^{V}Y_{v}^{2}+\sum_{v=1}^{V}P_{v}^{2}}\;+\;\sum_{v=1}^{V}Y_{v}logP_{v}\right)$$

### Performance evaluation metrics

This study employs the following metrics for quantitative analysis. IoU and DSC are core metrics that directly reflect the overlap of segmented regions, making them suitable for assessing the overall segmentation quality of augmented bone.^25^ Precision focuses on false positives and is applicable in scenarios sensitive to misdiagnosis. Sensitivity is incorporated to measure the ability of the model to identify all actual positive voxels, which ensures the comprehensive capture of the grafted material. HD95 was utilized to evaluate boundary fidelity and morphological accuracy in three-dimensional space. Accuracy is an auxiliary metric that should be analysed in conjunction with class balance.

To validate the consistency of the manual segmentation protocol and the stability of the trained model, we conducted reliability and reproducibility analyses on a randomly selected subset of 20 CBCT scans. These assessments are distinct from the primary ground truth creation process used for model training. To assess intra-observer reliability, one experienced surgeon re-segmented the augmented bone on 20 randomly selected CBCT scans after a 4-week interval to minimize recall bias. Reliability was quantified using the intraclass correlation coefficient (ICC) based on a two-way mixed-effects model for absolute agreement (ICC[A,1]), supplemented by Bland-Altman analysis.^26^^,^^27^ To assess inter-observer reliability, two trained surgeons independently segmented the augmented bone on the same 20 CBCT scans. Agreement was quantified using the same statistical metrics. To evaluate the technical reproducibility of the segmentation model, it was run twice on the same 20 CBCT scans under identical conditions. This procedure specifically assesses the model’s output stability and is distinct from the performance evaluation. Additionally, the time taken for manual segmentation by each of the two observers was recorded for 20 CBCT scans. This manual segmentation time was then compared with the computational time required for the model to generate segmentations for the same scans to evaluate relative efficiency.

### Visualization

The volume was computed in 3D Slicer by summing the volumes of all voxels encompassed by the segmentation mask. The segmented regions were subsequently exported as stereolithography(STL) files for visualization. Four representative CBCT images were selected and visualized using 3D Slicer, showcasing the coronal, sagittal, and axial planes. Surface distance heatmaps were generated to visually assess accuracy, comparing the 3D surfaces from deep learning predictions against the ground truth segmentations. The heatmap colour indicates point-to-surface distance (mm), with blue representing high agreement (distance ≈ 0 mm) according to the colour bar. Additionally, a representative case was selected to visualize the volumetric changes between the immediate and 6-month postoperative time points. A 3D visualization was created using Mimics version 21.0 by superimposing color-coded volumes.

### Statistical analysis

Statistical analysis was performed using SPSS software (version 27.0). Normality tests were conducted on all data using the Shapiro-Wilk test, with all data presented as mean ± SD. For data that did not follow a normal distribution, median and interquartile range (IQR) were used to describe the central tendency and dispersion. Differences between groups were compared using *t*-tests, Chi-square test, Kruskal-Wallis one-way ANOVA, Wilcoxon signed-rank test, and Mann-Whitney U test. The consistency of the volume derived from segmentation was assessed using the ICC.

## Results

### Model performance

The performance evaluation focused on four deep learning models: UNETR++, Swin Transformer, U-Net, and 3D-VNet. These models were compared using established segmentation performance indicators, specifically the DSC, IoU, sensitivity, precision, HD95, and accuracy. Among the tested models, UNETR++ demonstrated the best overall performance across these metrics. Detailed quantitative results for each model are presented in Table 2.

**Table 2:** Performance metrics of deep learning models.

Model	UNETR++	Swin Transformer	U-Net	3D-VNet
DSC	0.8477	0.7521	0.6542	0.7156
IoU	0.7356	0.6522	0.5288	0.5920
Sensitivity	0.8337	0.7486	0.6421	0.7117
Precision	0.8622	0.7556	0.6668	0.7195
HD95 (mm)	0.9234	1.1541	2.0567	1.4821
Accuracy	0.8730	0.7750	0.6864	0.7386

**Fig. 2:**
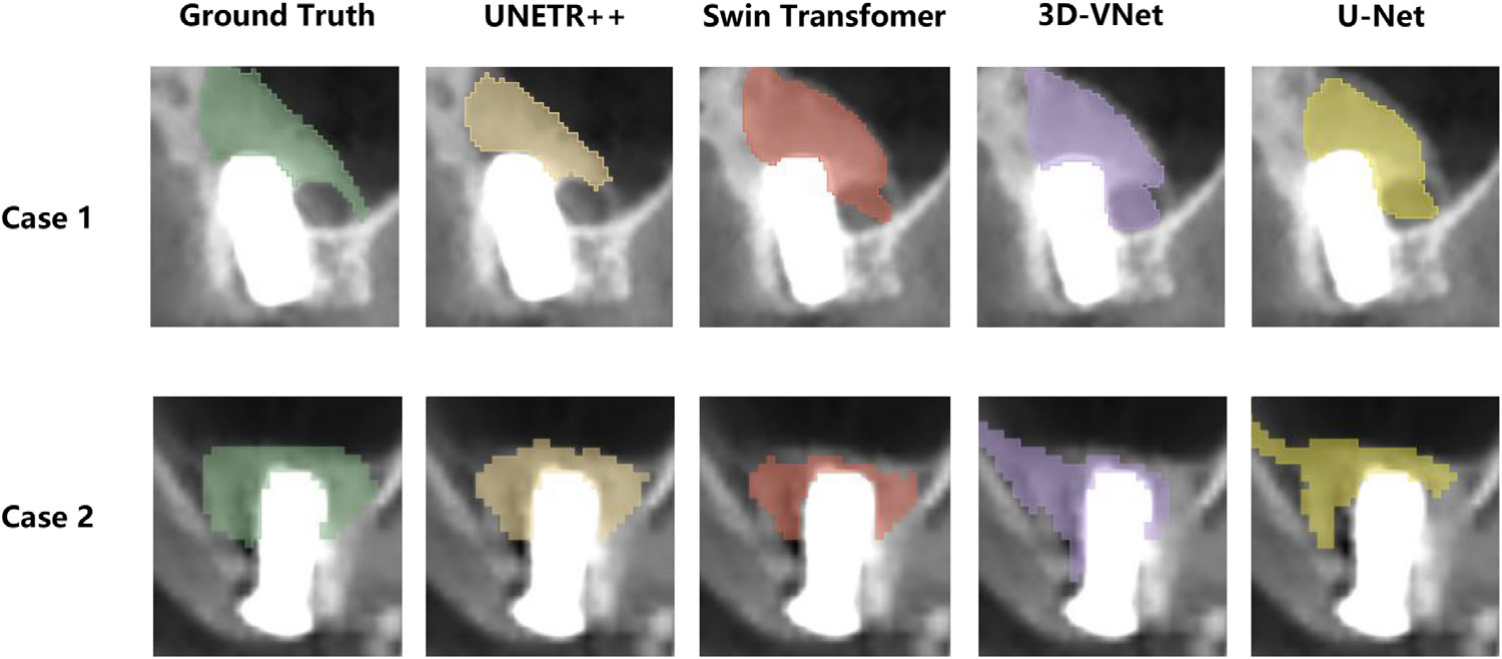
Qualitative comparison of segmentation performance on representative test cases.

Qualitative results of two representative test cases are presented in Figure 2. In the well-performing scenario characterized by clear boundaries, UNETR++ demonstrated superior capability in preserving intricate marginal details. Furthermore, in a challenging case complicated by irregular bone morphology and low contrast, while all models exhibited reduced performance, UNETR++ showed greater resilience.

This table presents the performance metrics of various deep learning models, including UNETR++, Swin Transformer, U-Net, and 3D-VNet. The comparison highlights differences in DSC, IoU, sensitivity, precision, HD95, and accuracy.

### Consistency and efficiency

**Fig. 3:**
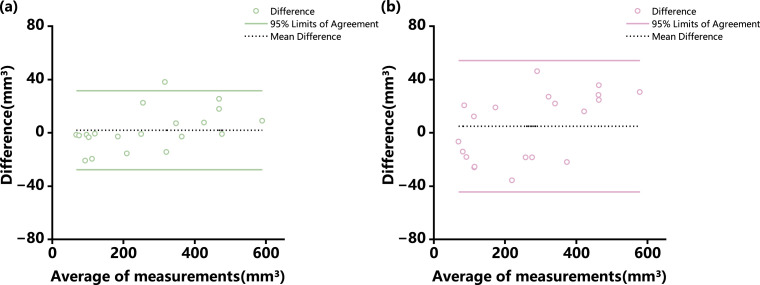
Bland-Altman analysis of observer reliability for volumetric measurements.

**Fig. 4:**
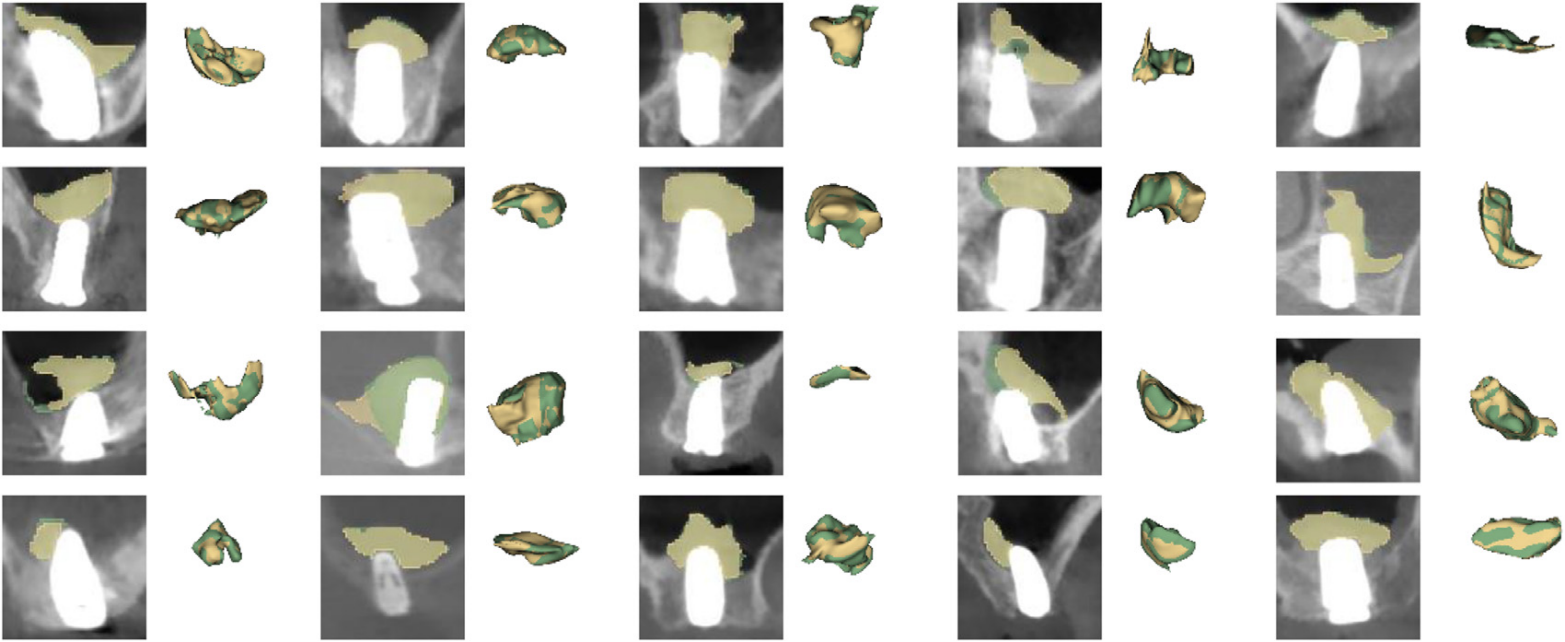
Visual assessment of intra-observer reliability for manual segmentation.

**Fig. 5:**
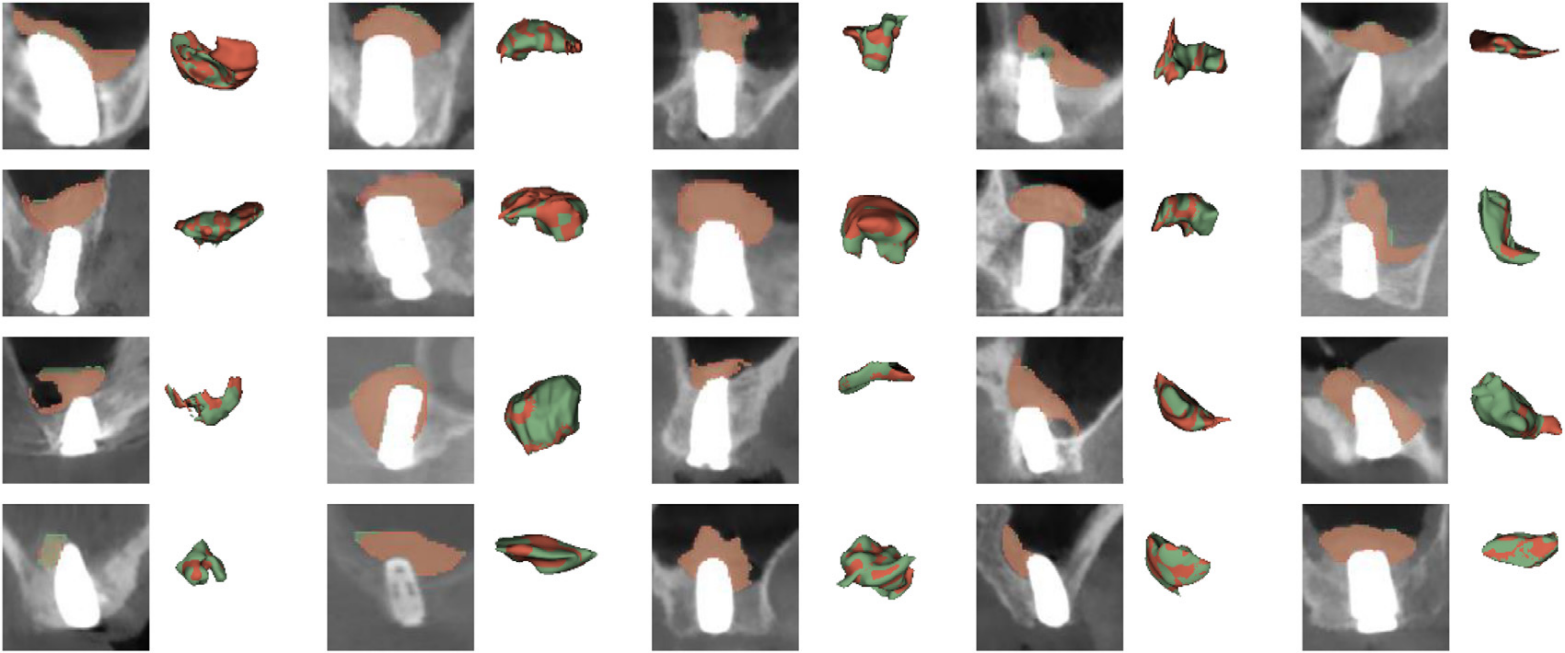
Visual assessment of inter-observer reliability for manual segmentation.

The consistency of manual segmentations and the reproducibility of the automated model were evaluated. The consistency of the manual segmentations demonstrated exceptionally high reliability, with ICC values for both intra- and inter-observer agreement exceeding 0.99. While these high ICC values confirm strong correlation, they do not reveal the magnitude or pattern of the differences. Therefore, Bland-Altman analysis was further employed to visually inspect the agreement in volumes derived from the segmentations and to assess for any potential systematic bias, either between different observers (inter-observer) or across repeated sessions by a single observer (intra-observer). The intra-observer analysis revealed mean difference (bias) of 1.90 mm³ and 95% limits of agreement (LoA) from −27.78 mm³ to 31.57 mm³ (Figures 3a and 4). For inter-observer reliability, the mean difference was 4.76 mm³ with 95% LoA of −44.54 mm³ to 54.06 mm³, indicating acceptable agreement (Figures 3b and 5). As the model is deterministic, it yields identical segmentation outputs for the same input image on every execution. Consequently, its reproducibility is perfect by definition, with repeated segmentations of the same scan yielded identical results, resulting in a mean difference of 0.00 mm³ and 95% LoA of 0 mm³. Furthermore, the automated model demonstrated remarkable efficiency. The average time for the model to complete the segmentation and volume calculation was only 14.96 ± 2.57 seconds. This was substantially faster than the manual segmentation times required by the two surgeons, which averaged 21.32 ± 1.34 minutes and 22.78 ± 1.68 minutes, respectively.

(a) Intra-observer reliability, assessing the agreement between two repeated measurements by the same observer. (b) Inter-observer reliability, assessing the agreement between measurements from two different observers. The dashed line represents the mean difference (bias), and the solid lines indicate the 95% limits of agreement.

Representative examples from the 20 repeated measurements are shown in both 2D slices and 3D reconstructions. The images compare an observer’s initial segmentation (green) with their repeated segmentation performed after a 4-week interval (yellowish) to visualize the variation between the two measurements.

Representative examples from the 20 scans are shown in both 2D slices and 3D reconstructions. The images compare the segmentations from two independent observers, with results from Observer 1 in green and Observer 2 in red, to illustrate the variation between them.

### Visualization

**Fig. 6:**
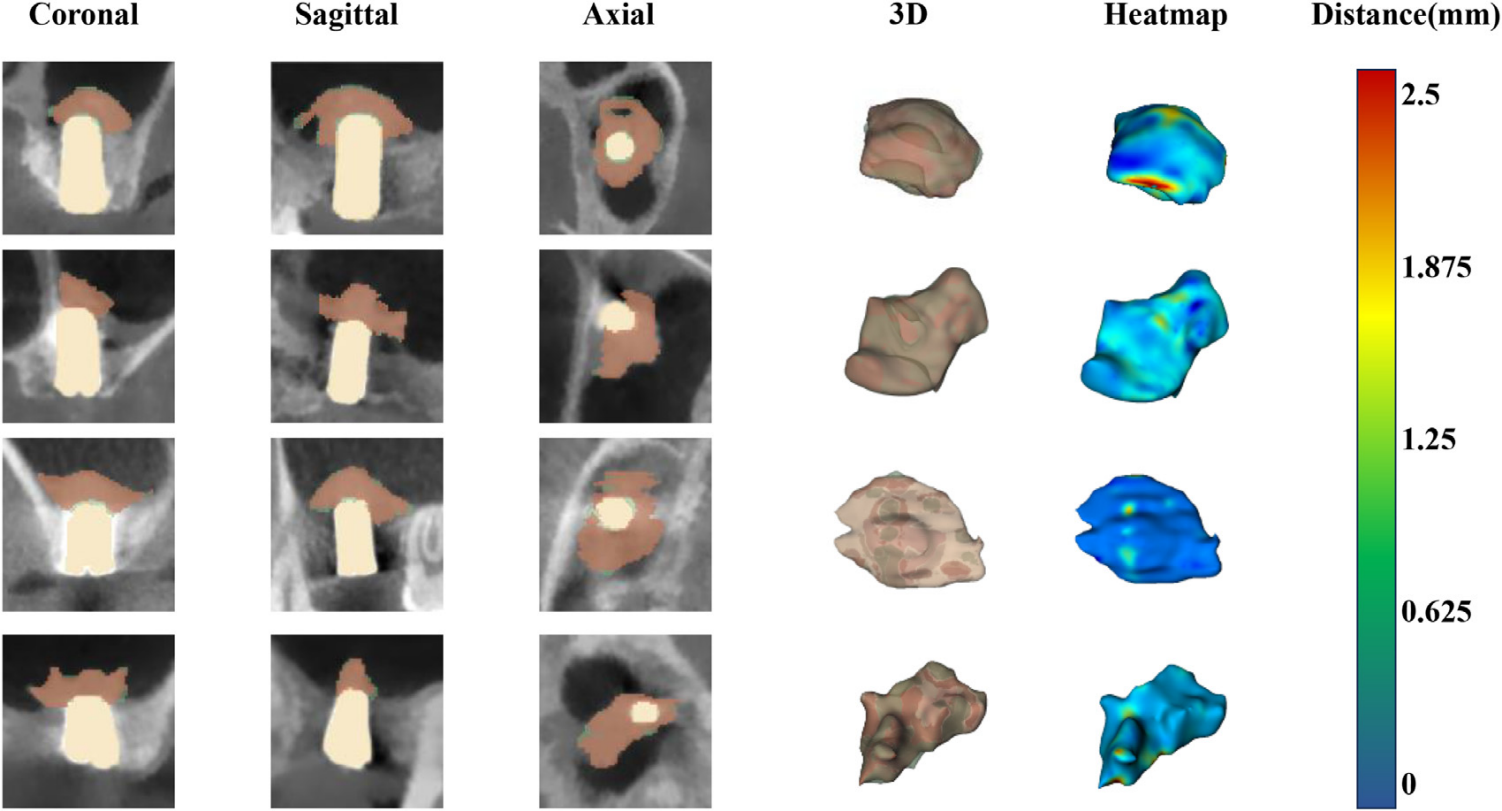
Visual comparison between model segmentations and ground truth.

Automated segmentations generated by the UNETR++ model were compared with the ground truth across multi-planar views (coronal, sagittal, and axial), as well as on 3D reconstructions of the augmented bone. A high degree of spatial overlap was observed between the model’s output and the ground truth, clearly illustrating the high accuracy of the model’s segmentation capabilities. Furthermore, heatmaps visualizing the surface distance between the model segmentations and the ground truth were predominantly characterized by blue regions, indicating minimal discrepancies (Figure 6).

Multi-planar (coronal, sagittal, axial) views from 3D reconstructed CBCT images. For both 2D slices and 3D reconstructions, red segments represent the model’s output, green segments represent the ground truth, and light yellow sections indicate the dental implants. The heatmaps display the surface distance between the model’s output and the ground truth. The colour bar represents the surface distance.

**Fig. 7:**
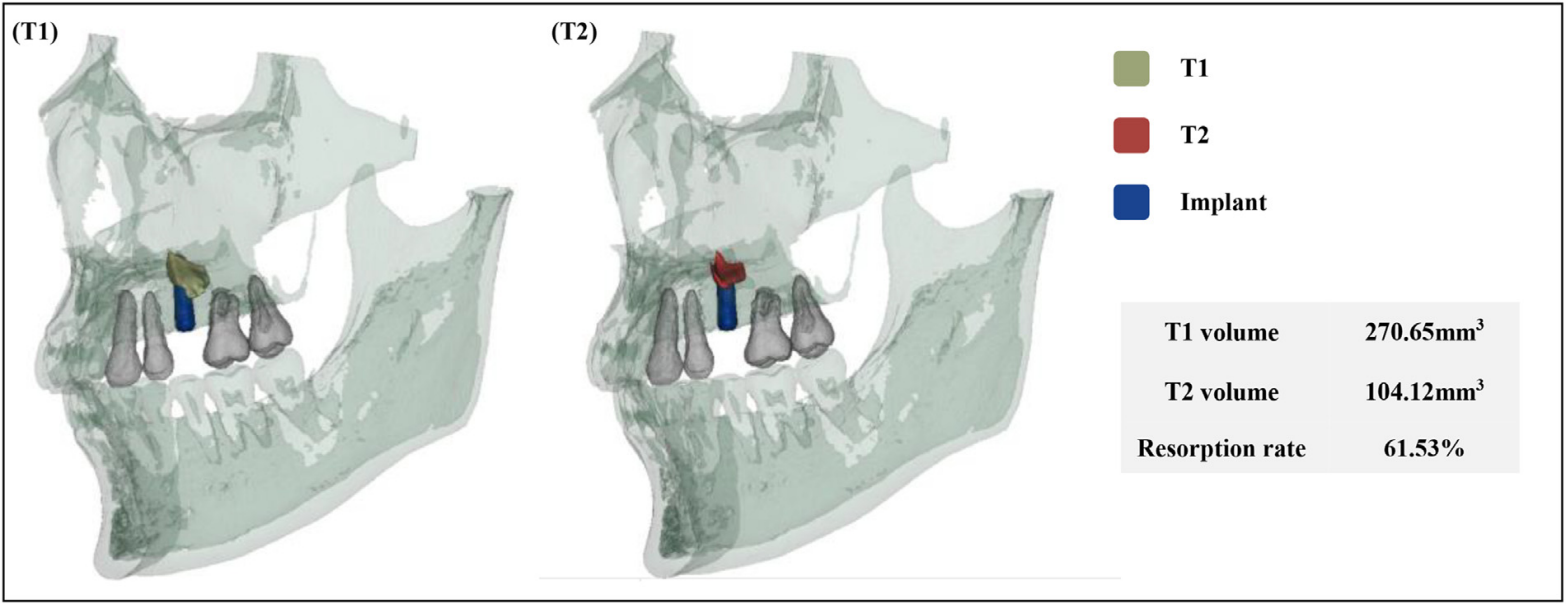
Visualization of augmented bone volume changes.

The green segment corresponds to the immediate(T1) augmented bone, the red segment represents 6-month postoperative(T2) augmented bone, and the blue segments indicate the implants. This allows for a direct visual comparison of the 3D bone morphology at different time points. By clearly illustrating the augmented bone volume changes, the visualization highlights the extent of bone resorption, particularly during the early postoperative period (Figure 7).

In the 3D reconstruction model, green segment represents the augmented bone at immediate(T1), red segment represents the augmented bone at 6-month postoperative(T2), and blue segments represent the implants. The reconstructed model shows that the augmented bone volume at T1 was 270.65 mm³, which decreased to 104.12 mm³ at T2, with resorption rate of 61.53%.

## Discussion

This study successfully implemented and validated deep learning models for the automated segmentation of augmented bone following TSFE, utilizing CBCT data.

In terms of segmentation, 3D-VNet demonstrated superior performance compared to the conventional U-Net. The enhanced performance of 3D-VNet can be attributed to its ability to incorporate volumetric information, which allows for more accurate segmentation of 3D structures.^17^ Transformer-based methods, such as UNETR++ and Swin Transformer, outperformed CNN-based methods in terms of segmentation accuracy. These models leverage self-attention mechanisms, which enable them to capture long-range dependencies and contextual information across the entire image.^28^ Specifically, the UNETR++ model showed the best performance across several metrics, including DSC, IoU, sensitivity, precision, HD95, and accuracy. The success of UNETR++ can be attributed to the combination of the transformer architecture with the U-Net structure, which allows for more effective feature extraction and spatial representation. The self-attention mechanism in transformer-based models enhances the ability to discern subtle details and refine segmentation boundaries, resulting in improved performance compared to traditional CNN-based approaches like U-Net.

Previous studies have predominantly focused on the segmentation and evaluation of augmented bone following LSFE.^29^ Due to the relatively regular morphology of grafted bone and the availability of abundant imaging data in LSFE cases, the technical implementation of such studies has been relatively mature.^8^ For instance, a recent study utilized a Swin Transformer-based model for LSFE segmentation and reported a high DSC of 0.8488 and an HD95 of 0.6464 mm. When applying the standard Swin Transformer backbone to our TSFE dataset, the performance was lower than that reported for LSFE, with a DSC of 0.7521 and an HD95 of 1.1541 mm. This performance gap underscores the significant challenges associated with TSFE segmentation. Primarily, the complex and irregular morphology of the augmented bone after TSFE complicates boundary delineation. Additionally, the mixture of autogenous bone with bone collagen further obscures the boundaries, making it difficult to accurately delineate the augmented bone area. This blending of materials results in varying densities and textures, which traditional imaging techniques may struggle to differentiate. Consequently, advanced imaging methods and sophisticated segmentation algorithms are necessary to achieve precise and reliable evaluations of augmented bone volume following TSFE.

By applying deep learning models, this study provides a novel solution for the segmentation of augmented bone following TSFE, showing excellent performance. Compared with traditional imaging segmentation methods, such as manual measurements based on 2D slices derived from CBCT images, deep learning demonstrates significant advantages in terms of stability and time efficiency. This technical advantage not only enhances segmentation accuracy but also significantly reduces the need for manual intervention, thereby improving the reproducibility and reliability of segmentation results. Furthermore, the high-resolution segmentation capability of deep learning lays a solid foundation for in-depth analysis of postoperative bone integration. This allows clinicians to gain a more reliable quantitative understanding of bone augmentation outcomes, particularly the spatial and temporal characteristics of bone resorption. This detailed insight into the dynamics of bone healing is crucial for clinical evaluation and decision-making in implantology. It helps clinicians assess augmented bone stability, predict long-term treatment outcomes, and ultimately, can guide the optimization of surgical techniques to improve patient care.^30^

Despite its promising results, this study has certain limitations. Firstly, while UNETR++ shows excellent segmentation accuracy and efficiency, its performance is highly dependent on the quality of CBCT imaging data. Variations in imaging quality, such as noise, artifacts, or insufficient resolution, can negatively impact the stability and reliability of segmentation results, necessitating further optimization and validation in clinical practice.^31^ Additionally, factors specific to bone augmentation procedures, such as membrane thickness and the presence of dental implants, can introduce significant artifacts in CBCT images. Thicker membranes may be more difficult to distinguish from surrounding tissues, while metallic implants create scatter artifacts that can obscure adjacent structures and compromise segmentation accuracy in those regions.^32^ The selection of deep learning models in this study presents certain limitations. Given that the compared architectures span different stages of algorithmic advancement, the superior performance of recent models is largely anticipated. Future research should evaluate a broader spectrum of architectures, including emerging AI foundation models, to further validate the applicability and robustness of advanced architectures in TSFE segmentation. The spatial resolution of CBCT scans is an additional factor that warrants consideration. In this study, voxel sizes ranged from 0.25 mm to 0.35 mm, with 0.30 mm being the most prevalent resolution. Although some research suggests that voxel size may influence the trueness of segmentation, the retrospective nature and data imbalance of our cohort prevent a definitive assessment of its impact on the model.^33^ In particular, the disproportionate distribution of different resolutions limits the statistical power required to isolate voxel size as a primary variable. Previous evidence indicates that while finer resolutions may provide theoretical benefits, actual gains in volumetric precision are often contingent upon the anatomical complexity and the specific software utilized.^34^ Furthermore, the study’s sample size is relatively small and derived from a single-centre dataset, which may introduce selection bias.^35^ Future prospective studies with standardized follow-up protocols are necessary to validate these preliminary findings.

Consequently, future studies should validate the model using larger, multicentre datasets to assess its generalizability and applicability, ensuring consistent performance across diverse patient populations and clinical scenarios.^36^ Beyond validation, it is also essential to correlate predicted volumetric changes with clinical metrics such as implant stability quotient (ISQ) values and long-term implant survival rates.^37^ Furthermore, future prospective studies should focus on optimizing voxel size parameters to achieve a critical balance between high segmentation precision and the reduction of patient radiation dose. This direction will be essential for establishing standardized scanning protocols that support both diagnostic reliability and patient safety. Crucially, the development of user-friendly software interfaces would then be vital for translating these capabilities into accessible clinical decision support tools. Such models could provide clinicians with more intuitive and quantitative predictions of postoperative bone resorption trajectories, aiding in the formulation of individualized treatment strategies and optimizing long-term treatment outcomes for patients.

## Conclusion

This study demonstrates that deep learning models, specifically the UNETR++ based segmentation approach, exhibit high accuracy and efficiency in the automatic segmentation of TSFE. Compared to traditional manual segmentation methods, these models significantly reduce operation time and improve segmentation efficiency. The automatically generated augmented volume data provide valuable support for clinical surgical planning, postoperative evaluation, and long-term follow-up, while also enhancing clinical workflow efficiency and the overall quality of diagnosis and treatment.

**Clinical significance**

Deep learning models accurately and efficiently segment augmented bone after TSFE, reducing surgeon variability and time. This tool has the potential to support improved treatment planning, postoperative assessment, and long-term follow-up.

Alt-text: Unlabelled box dummy alt text

## Author contributions

Conceptualization, Investigation, Writing-Original Draft: Kexin Yang. Data Curation, Formal Analysis, Visualization: Wenjun Duan. Methodology, Validation, Writing – Review & Editing: Wangtao Lu. Validation, Data Curation: Zheyuan Sun. Resources, Investigation: Rongan Li. Funding Acquisition, Project Administration, Interpretation: Jiakang Yang. Supervision, Writing – Review & Editing, Correspondence: Baixiang Wang.

## Data availability

The data that support the findings of this study are available on request from the corresponding author. The data are not publicly available due to privacy or ethical restrictions.

## Conflict of interest

The authors declare that they have no known competing financial interests or personal relationships that could have appeared to influence the work reported in this paper.

## Appendix Supplementary materials

Image, application 1
