# Supplementary material for: Automated Segmentation of Augmented Bone After Transalveolar Sinus Floor Elevation Using Deep Learning
Source: Int Dent J. 2026 Mar 6;76(3):109468. doi: 10.1016/j.identj.2026.109468 (PMC13147979; doi:10.1016/j.identj.2026.109468)
Supplement: Supplementary file 1 [file mmc1.doc]

#### **Supplementary material**

**Figure S1** U-Net architecture

Diagram illustrating the U-Net model used. It features an encoder path (left), a decoder path (right), and skip connections to merge feature maps across paths.

**Figure S2** 3D-VNet architecture

The V-Net model utilizes 3D convolutions in an encoder-decoder structure. Key features include residual connections within stages and skip connections linking the encoder (left) and decoder (right) paths.

**Figure S3** Swin Transformer architecture

Key components include patch partition, linear embedding, sequential stages containing Swin Transformer Blocks (using shifted window attention), and patch merging layers to create a hierarchical feature map from the input ROI.
